# Antibody–Drug Conjugates: Ushering in a New Era of Cancer Therapy

**DOI:** 10.3390/pharmaceutics15082017

**Published:** 2023-07-26

**Authors:** Joshua Hurwitz, Lucy Roxana Haggstrom, Elgene Lim

**Affiliations:** 1 St. Vincent’s Clinical School, Faculty of Medicine and Health, University of New South Wales, Kensington, NSW 2053, Australia; 2 Garvan Institute of Medical Research, Darlinghurst, NSW 2010, Australia; 3 Prince of Wales Hospital, Randwick, NSW 2031, Australia

**Keywords:** antibody–drug conjugate, cancer, clinical trials

## Abstract

Antibody–drug conjugates (ADCs) have provided new therapeutic options and significant promise for patients with cancer, particularly where existing treatments are limited. Substantial effort in ADC development is underway globally, with 13 ADCs currently approved and many more in development. The therapeutic benefits of ADCs leverage the ability to selectively target cancer cells through antibody binding, resultant relative sparing of non-malignant tissues, and the targeted delivery of a cytotoxic payload. Consequently, this drug class has demonstrated activity in multiple malignancies refractory to standard therapeutic options. Despite this, limitations exist, including narrow therapeutic windows, unique toxicity profiles, development of therapeutic resistance, and appropriate biomarker selection. This review will describe the development of ADCs, their mechanisms of action, pivotal trials, and approved indications and identify common themes. Current challenges and opportunities will be discussed for this drug class in cancer therapeutics at a time when significant developments in antibody therapies, immunotherapy, and targeted agents are occurring.

## 1. Introduction

The search for directed and effective cytotoxic therapy has been the Holy Grail of cancer treatment since being hypothesised as a treatment for cancer over a century ago [1]. Despite this, the mainstay of treatment for over half a century and up until today remains chemotherapy. While effective in many cancers, chemotherapy is associated with frequent off-target effects resulting in significant toxicities [2]. Antibody–drug conjugates (ADCs) are compounds comprising three distinct components to effectively target cancer cells: a monoclonal antibody, a cytotoxic payload, and a linker that binds these two components. ADCs have been successful as they selectively target cancer cells with a highly toxic payload via antibody binding to specific tumour antigens, and spare non-malignant tissues [3,4].

The theory behind ADCs was first developed in the 1960s, with the first animal studies conducted in the 1980s [5]. Challenges with the first generation of ADCs included the unstable linker component, which led to premature drug release into the circulation [6]. Stabilising the drug molecule, determining appropriate ratios of drug to antibody, and improving ADC half-lives have been major challenges in ADC development [7]. Conventional chemotherapies such as anthracyclines were originally used as payloads but were ineffective due to a lack of relative lack of potency [6]. Early antibodies used were predominantly chimeric humanised or mouse-derived and were associated with higher immunogenicity and drug reactions compared to next-generation humanised antibodies [6]. Another barrier to the utility of ADCs was the limited number of antigen targets. Significant progress has been made since and a growing number of ADCs have now been approved by the Food and Drug Administration (FDA) for clinical use following positive results from phase 3 trials when compared against current standards of care. In this review, we will discuss the structure and mechanisms of actions of ADCs, present the data from seminal clinical ADC trials, and provide an overview of the challenges facing the ongoing clinical development of ADCs.

## 2. ADC Structure and Mechanism of Action

**Figure 1:**
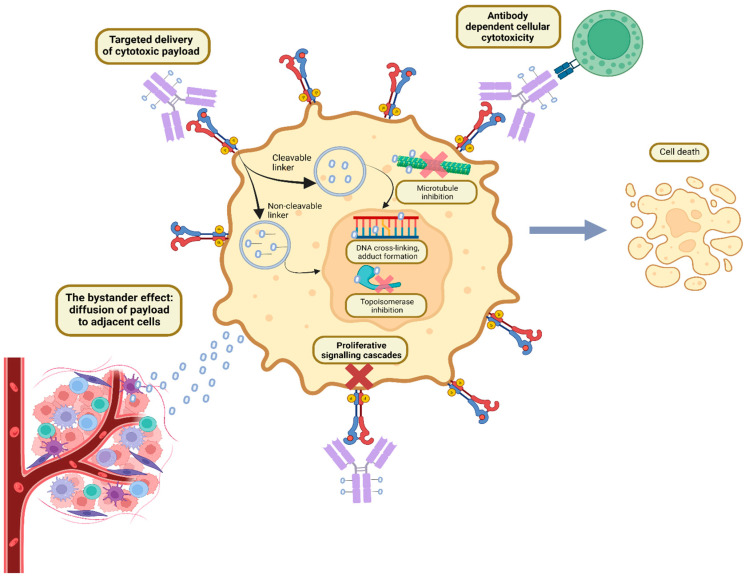
Mechanism of action of antibody–drug conjugates. Created with BioRender.com.

**Figure 2:**
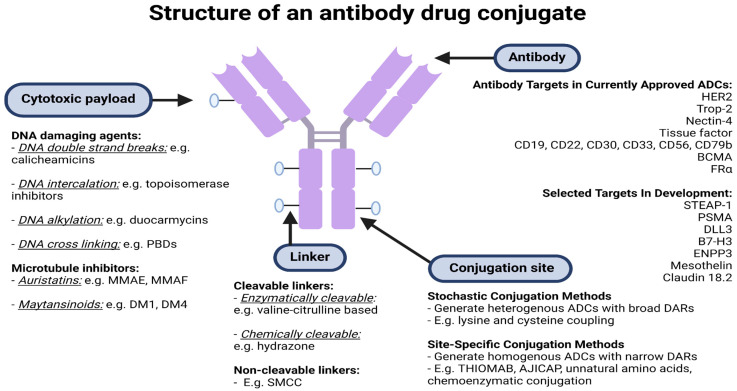
**Structure of an antibody–drug conjugate. Abbreviations:** BCMA, B-cell maturation antigen; B7-H3, B7 homolog 3 protein; DAR, drug to antibody ratio; DLL3, deltalike protein 3; ENPP3, ectonucleotide pyrophosphatase/phosphodiesterase family member 3; FRα, folate receptor α; MMAE/F, monomethyl auristatin E/F; SMCC, succinimidyl-4-(N-maleimidomethyl) cyclohexane-1-carboxylate; PBD, pyrrolobenzodiazepine; PSMA, prostate specific membrane antigen. Created using BioRender.com.

ADCs have a unique formulation consisting of three primary components: an antibody, cytotoxic payload bound by a chemical linker, as illustrated in Figure 1 and Figure 2. Each plays its own role in targeting cancer, efficacious delivery, and a desired cytotoxic outcome [8]. 

### 2.1. Antibodies and Target Antigens

Monoclonal antibodies, while used for cancer treatment for many years, have alone not provided the desired outcome regarding therapeutic benefit in most cancers. As such, the vast majority of cancer treatment protocols which include antibodies are given in combination with chemotherapy. ADCs represent a rational approach to harnessing the characteristics of both therapeutic classes [9]. Ideal target antigens are selectively over-expressed on cancerous cells and minimally on non-malignant tissues, which improves efficacy and limits toxicity [6]. However, most antigens are ‘tumour associated’, rather than ‘tumour-specific’, meaning there is still some expression of the antigen on non-malignant cells [10]. Antigens are required to be extracellular or on the cell surface, such as Trop-2 and HER-2, as intracellular antigens may not be recognised by the antibody [6]. In addition, the antigen should be displayed rather than secreted, as the latter can lead to ADC binding in the systemic circulation, increasing the risk of systemic toxicity [6,11]. 

The efficiency of ADCs depends on how effectively ADCs are internalised following binding to a target antigen, and on how they are internally processed [10]. For instance, the recycling of antigen–ADC complexes to the cell surface after internalisation compromises ADC efficacy, compared to those that are not recycled and instead undergo lysosomal processing and payload release [10]. Antigen shedding, which is a process whereby antigens are removed from the cell surface, also limits the efficacy of ADCs [10]. The internalisation efficiency of the target antigen is another important factor that influences ADC efficacy. ADCs that are rapidly internalised penetrate solid tumours less than ADCs that are slowly internalised [12]. Similarly, ADCs that have a very high affinity to antigens often bind predominantly to malignant cells in perivascular regions and have lower diffusion compared to ADCs that have lower binding affinity [12]. The internalisation rate of antigen–antibody complexes is complex and influenced by a wide variety of factors, including the co-expression of other cell surface receptors, the degree of expression of the antigen, and the binding affinity of the antigen [12,13]. For instance, CD19-targeted ADCs can be efficiently endocytosed, but only in the absence of expression of CD21 [14]. As such, the optimal internalisation kinetics are highly tumour- and drug-specific.

Current ADCs involve antibodies that are fully humanised, reducing immunogenicity [6]. Most ADCs are based on immunoglobulin G (IgG) antibodies, which have four known subtypes. The benefits of IgG1, the most common subtype used, include its ability to induce antibody-dependent cell-mediated cytotoxicity, complement-dependent cytotoxicity, and antibody-dependent phagocytosis [6]. 

### 2.2. The Payload

The second part of any ADC is the cytotoxic payload, commonly termed the ‘missile’ or ‘warhead’ in the existing literature. With only 2% of ADCs reaching target tumour cells [15], potency is essential for these compounds to be efficacious [6,16]. Most agents approved today have employed two categories of payloads, DNA damaging agents and microtubule inhibitors [6]. DNA damaging agents include agents which lead to DNA double-stranded breaks (e.g., calicheamicins), DNA intercalation (e.g., topoisomerase inhibitors), DNA alkylation (e.g., duocarmycins), and DNA cross-linking (e.g., pyrrolobenzodiazepines) [6]. These potent DNA-damaging agents have an IC_50_ in the picomolar range [6,16]. Microtubules are a key cytoskeletal element that play an important role in cell division [6]. Microtubule inhibitors include auristatin derivatives, such as monomethyl auristatin E and F (MMAE and MMAF), and maytansinoid derivatives, such as DM1 and DM4 [6]. They typically possess IC_50_ values in the nanomolar range [6]. 

The drug–antibody ratio (DAR), defined as the number of payload molecules that can be attached to the antibody, influences the potency and therapeutic index of ADCs [17]. The DAR also impacts the physiological properties of binding, the drug’s pharmacokinetics, and its half-life [18]. Most currently approved ADCs have a DAR ranging from 2–8 [6]. 

### 2.3. Linkers

Linkers play a critical role in ensuring ADC stability and in optimising the delivery of the cytotoxic payload to tumour cells. Optimal linkers are stable in the circulation, which prevents premature payload release and systemic toxicities, but then rapidly cleaved once internalised in malignant cells, promoting efficient cell death [19,20]. There are two types of linkers, classified based on the payload release mechanism: cleavable and non-cleavable [21]. Cleavable linkers are designed to be degraded intracellularly, in response to a change from the extracellular environment to the intracellular environment [20]. There are a variety of triggers for the degradation of cleavable linkers, including specific lysosomal proteases such as cathepsin B (which cleaves Val-Cit and Val-Ala linkers coupled with PABC), acidic pH (which cleaves hydrazone), and glutathione (which cleaves disulfide bonds) [20]. The majority of currently approved ADCs use cleavable linkers, such as trastuzumab deruxtecan (T-DXd) and inotuzumab ozogamicin [6,21]. The advantages of cleavable linkers are the intracellular release of the payload and stability in the circulation, while disadvantages include potential premature cleavage in the peripheral circulation [20,22,23,24]. 

In contrast, non-cleavable linkers consist of stable bonds that are resistant to proteolysis [21]. ADCs containing non-cleavable linkers rely on the near complete degradation of the antibody component by cytosolic and lysosomal proteases, which leaves the payload attached to the linker and an amino acid residue derived from the antibody [21]. Examples of ADCs containing non-cleavable linkers include trastuzumab emtansine (T-DM1), which contains N-succinimidyl-4-(N-maleimidomethyl) cyclohexane-1-carboxylate (SMCC) and belantamab mafodotin, which contains a maleimidocaproyl linker [21,25]. Potential advantages of non-cleavable linkers are in limiting off-target toxicities occurring from premature payload release; however, the disadvantages are that an amino acid residue remains attached to the payload, which can influence the pharmacokinetics and pharmacodynamics of the payload [20]. 

### 2.4. Conjugation Chemistry

Optimising conjugation chemistry is critical in improving ADC efficacy. Chemical conjugation and enzymatic conjugation are the two main traditional stochastic methods used to bind antibodies to their payloads [20]. Chemical conjugation involves a reaction between amino acid residues on the antibody and a reaction site on the linker [20]. Examples of chemical conjugation methods include lysine amide coupling (used in T-DM1), and cysteine coupling, where the payload is bound to lysine or cysteine residues on the antibody, respectively [20]. These methods typically generate heterogeneous ADC species with variable DARs, which are as suboptimal as ADCs with broad DAR distributions and are less efficacious than those with narrow DAR distributions [20,26]. For instance, on a typical antibody, there are approximately 10 chemically accessible lysine residues; hence, lysine coupling can lead to variable ADC species with a broad DAR distribution [27]. Cysteine-based coupling involves a reaction between reduced interchain cysteine residues on the antibody and thiol groups on the payload, and is superior to lysine coupling, as the number of conjugation sites is more limited, generating ADCs with more homogeneous DARs [20].

Another key limitation of traditional coupling methods is that the site of the payload attachment to the antibody is stochastically distributed [28]. This stochastic distribution leads to unpredictable pharmacokinetic effects, as for example, binding of the payload to sites on the antibody that participate in antigen binding can substantially alter the pharmacokinetics and biological activity of the ADC [28]. In vitro assays and pharmacokinetic analyses in xenograft models have introduced cysteine conjugation at various antibody positions and compared this to enzymatic conjugation using microbial transglutaminase on the light chain or heavy chain [29]. ADCs produced using enzymatic conjugation to the light chain or position Q295 on the antibody had superior pharmacokinetic behaviour, as did those engineered with cystine conjugation to the L328 position [29]. This research highlights the differences in pharmacokinetic profiles that can occur with different conjugation sites [29]. 

Site-specific conjugation methods have several advantages over classical conjugation methods, namely, improved ADC homogeneity and binding of the payload to an antibody region that does not participate in antigen binding [28,30]. Site-specific conjugation has now become the primary method of conjugation used since 2020 [30]. Several methods of site-specific conjugation exist. THIOMAB was one of the first site-specific conjugation methods developed and is an engineered cysteine-based technology that enables the production of highly homogeneous ADCs with a DAR of 2 [31]. Limitations of THIOMAB technology include a limited DAR and the use of genetic engineering, which can be costly and time-consuming [20]. The use of unnatural amino acids is another chemical site-specific technique. This involves engineering antibodies that contain unnatural amino acids, which subsequently react with linkers, leading to tightly controlled DAR and homogenous ADCs [20]. However, these benefits are at the expense of significant cost and potential undesired immunogenicity due to the unnatural amino acid [20]. Another chemical site-specific technique is the ‘AJICAP’ technology, which involves introducing thiol functional groups onto three lysine residues on IgGs using peptide reagents and avoids the need for antibody engineering [28,32,33]. The first-generation AJICAP technique had several limitations including the need for lengthy reduction and oxidation reactions and aggregation of a small proportion of the generated ADCs [34]. These issues have been improved in the newly developed second-generation AJICAP technology, which enables production of a wide variety of ADCs with homogenous DARs of 2 and improved therapeutic index [34]. Various methods of analysing ADCs exist, including hydrophobic interaction chromatography, reversed-phase liquid chromatography, and size-exclusion chromatography, among others [35]. These techniques have various limitations in analysing traditional stochastic ADCs, owing to the ADC heterogeneity and broad DAR distribution [35]. The use of site-specific technologies can improve the accuracy of ADC analysis [35]. 

Chemoenzymatic conjugation methods employ enzymes, such as sortase, transglutaminase, and glycosyltransferase, to bind a linker to the antibody in a site-specific manner. Enzymatic approaches can avoid the need for costly and time-consuming antibody engineering, but challenges with enzymatic approaches include cost, difficulties in large-scale enzyme production, and difficulties in removing the enzyme from the conjugation reaction matrix [26,32]. The type of immunoglobulin used also influences conjugation efficiency. While IgG1 is the most common antibody used, IgG2 theoretically offers more conjugation sites, which may increase ADC potency [36,37]. 

### 2.5. ADC Purification

There are several steps involved in ADC production. In brief, the first process involves the monoclonal antibody being reconstituted in a buffer stabilising the antibody. The next phase is removing small molecules and providing a solution suitable for the conjugation reaction to occur. Antibody modification is followed by conjugation where a crude ADC is formed. Following this, the crude ADC is purified with either single or sequential purification with a combination of chromatography and tangential flow filtration (TFF) [38]. Purification of ADCs leads to higher ADC concentration and improved pharmacokinetics [39]. 

Various methods of purification of ADCs exist. The first is TFF, a widely adopted technique that removes solvents, small molecular impurities, and drug-linker impurities. Limitations of this technique are that aggregates cannot be removed and remain in the final ADC, and various DAR species are unable to be separated [32]. Elevated DAR species lead to aggregation of the ADC product and, hence, higher in vivo clearance. The outcome of this is lower efficiency and safety, while low DAR species exhibit problems with efficacy [40,41,42]. Monoclonal antibodies in cell culture can be purified by chromatography. Manufacturing antibodies can be achieved via affinity chromatography and done at scale. This technique separates proteins, which assists in the analysis of ADCs, characterisation, along with separation and purification [43]. Multiple types of chromatography exist including size exclusion chromatography, hydrophobic interaction chromatography, ion exchange chromatography, and hydroxyapatite chromatography. Size exclusion chromatography uses molecular sizes to separate proteins while hydrophobic interaction chromatography assists in DAR determination using different hydrophobicity in native conditions to assist in DAR analysis. Limitations of this technique include low recoveries and potential antibody aggregation [44,45]. 

## 3. Pharmacokinetics and Pharmacodynamics of ADCs

ADCs are administered intravenously and can travel through the systemic circulation without being metabolised, essentially remaining inactive [46]. Its metabolism and elimination are crucial in ensuring drug delivery and excretion. Metabolism in circulation can influence the pharmacokinetics, efficacy, and toxicity profile [47]. The distribution of the ADC initially relies on “volume of distribution” due to vascular and interstitial space. It can also be impacted by antigen expression and subsequent internalisation of the ADC once the target is reached. Distribution can result in off-target toxicities if interacting with non-target tissues [9]. The metabolism of ADCs is complex and involves conjugation sites, linkers, and the payload. Chemical uncoupling through deconjugation or linker cleaving via enzymes are the commonest ways to release the drug into the plasma [47,48]. The internalisation or mobilisation of ADC leads to fusion with lysosomes, where cleavage of the linker occurs. This represents a mechanism of ADC elimination and clearance from circulation by receptor-mediated endocytosis with subsequent lysosomal compartment degradation. The cytotoxic payload is then free to reach the target, binding and triggering the death of the target cell. The free drug, if released into the cytosol, has been reported to travel across plasma membranes leading to a bystander effect where surrounding cells are also exposed to the cytotoxic payload. One upside of this is that a lower antigen expression on tumour cells is required allowing for the targeting of a larger population of cancerous cells, not only being limited to tumour cells with the highest target antigen expression [49].

Proteolytic degradation or catabolism assists in eliminating the monoclonal antibody portion of the ADC, which is repurposed as a protein or new carbon source. They cannot be excreted by the liver or exit the systemic circulation through glomerular filtration. In contrast, the excretion of the payload occurs through the renal and hepatic organs and varies with the ADCs [50]. For example, the payload of T-DM1 is predominantly excreted via the hepatic system with minimal renal excretion, while brentuximab vedotin is predominantly excreted in faeces via the hepatic route [51]. There is limited published data with newer ADCs like sacituzumab govitecan on the elimination process and its impact on hepatic or renal function [52]. 

### Bystander Effect

The bystander effect is an increasingly recognised mechanism of ADCs. This occurs where cells within close proximity of the targeted cancer cells are exposed to the antitumour effects of ADCs, irrespective of antigen expression [53]. The bystander effect depends primarily on the nature of the linker and the payload. ADCs with cleavable linkers and hydrophobic payloads have been shown in preclinical models to diffuse through cell membranes and elicit the bystander effect [54]. The impact of the payload and linker on the ability to elicit the bystander effect is illustrated by comparing the mechanism of two similar ADCs with a trastuzumab antibody, T-DXd and T-DM1 [55,56]. T-DM1 consists of a non-cleavable linker, and after drug internalisation into antigen-positive cells, trastuzumab is degraded while a peptide chain from the linker remains bound to emtansine. This compound is charged at physiological pH and, hence, remains within the cells and does not diffuse to surrounding cells. Therefore, the bystander effect is limited with T-DM1 [6]. However, ADCs using trastuzumab and emtansine, but with a cleavable linker have been created, and can induce the bystander effect, as after internalisation, the linker is degraded, leaving the hydrophobic maytansinoid, which can diffuse into surrounding cells [57].

In contrast, T-DXd consists of a cleavable linker, and after internalisation of T-DXd in antigen-positive cells, the linker is degraded, leaving the hydrophobic payload deruxtecan. This hydrophobic payload is able to diffuse to neighbouring antigen-negative cells, inducing the bystander effect [56]. Furthermore, increasing the hydrophobicity of payloads, for example, by adding more methylene groups to maytansinoids, has been shown to increase bystander killing [58]. Similar to T-DXd, trastuzumab duocarmazine is another ADC that demonstrates a significant bystander effect. Trastuzumab duocarmazine is also a cleavable linker with a duocarmycin payload [59]. Other approved ADCs have also demonstrated the bystander effect in preclinical models including enfortumab vedotin (EV), tisotumab vedotin (TV), and sacituzumab govitecan (SG) [54]. MMAE and MMAF, which are extracted from sea hares, are commonly used cytotoxic payloads. MMAE exhibits bystander effect properties as it is not bound by cell membranes, while MMAF does not, and has thus been found to be less efficient and less toxic due to this pivotal difference [60,61]. While specific research in the bystander effect in the haematological space is limited, MMAE conjugates brentuximab vedotin and polatuzumab vedotin have been approved, respectively, for use in Hodgkin lymphoma and refractory diffuse large B-cell lymphoma. Brentuximab vedotin has also demonstrated the bystander effect in germ cell tumours expressing CD 30 positive and negative cells [62]. As MMAE-based ADCs often utilise cleavable linkers known to retain membrane permeability, these characteristics underpin their ability to create a bystander effect [63].

## 4. Seminal Phase II/III Trials of Antibody–Drug Conjugates in Cancer

There are currently 13 ADCs approved by the US Food and Drug Administration (FDA) for various malignancies, with approvals occurring particularly rapidly since 2017 [6]. Here, we summarise the pivotal trials of ADCs in solid and haematologic malignancies (illustrated in Table 1 and Table 2).

### 4.1. Trials of ADCs in Solid Organ Malignancies

Three ADCs have been FDA-approved for the treatment of breast cancer. This is not surprising, as therapeutic antibodies are well established in treating this disease, and ADCs represent an extension of this approach. T-DM1 was the first ADC approved for breast cancer and consists of a humanised HER2-directed monoclonal antibody trastuzumab, linked via a non-cleavable linker to DM1, a microtubule inhibitor, with a DAR of approximately 3.5 [6]. It has been shown to improve median overall survival in patients with metastatic HER2-positive breast cancer treated in the second-line setting compared to capecitabine plus lapatinib, with a hazard ratio of 0.68 (95% CI 0.55 to 0.85, *p* < 0.001) [64]. It has also been approved for use in patients with residual HER2-amplified breast cancer after neoadjuvant HER2-directed therapy and chemotherapy, where it has been shown to improve invasive disease-free survival by 50% compared to adjuvant trastuzumab (HR 0.50, 95% CI 0.39 to 0.64; *p* < 0.001) [65]. 

T-DXd has subsequently been shown to improve outcomes compared to T-DM1. T-DXd is a newer ADC that consists of the same HER2-directed monoclonal antibody trastuzumab, linked via a cleavable tetrapeptide linker to the payload deruxtecan, which is a topoisomerase 1 inhibitor [6]. The DESTINY-Breast03 trial, compared to T-DXd to T-DM1 in patients with metastatic HER2-positive breast cancer in the second-line setting, reported an impressive hazard ratio of 0.28 for disease progression or death (95% CI 0.22 to 0.35, *p* < 0.001) [66]. Furthermore, the DESTINY-Breast02 trial is the first and only trial exploring ADC use in patients who have previously progressed on another ADC, T-DM1. It showed that T-DXd was superior to the treatment of physician’s choice in patients with metastatic HER2-positive breast cancer previously treated with T-DM1, with a hazard ratio of 0.36 for progression-free survival (PFS) (95% CI 0.28 to 0.45; *p* < 0.0001) [67]. 

Historically, HER2-positive breast cancer has been defined in a binary fashion as either HER2 positive, defined as a score of 3+ on immunohistochemistry (IHC) or 2+ on IHC and positive in situ hybridisation (ISH), or HER2 negative, defined by a score of 0–1 on IHC or 2+ on IHC and negative on ISH [68]. However, in the phase III randomised controlled DESTINY-Breast04 trial, T-DXd was shown to have benefits in patients with ‘HER2 low’ breast cancer, defined as a score of 1+ on IHC or as 2+ on IHC with negative ISH, with a hazard ratio of 0.64 for overall survival (95% CI 0.49 to 0.84) [68]. This pivotal study has redefined the treatment algorithm and classification of breast cancer. In terms of toxicity, drug-related interstitial lung disease or pneumonitis occurred in 12.1% of patients receiving T-DXd on the DESTINY-Breast 04 trial, including 3 (0.8%) with a fatal event. In the DESTINY-Breast 03 trial, 10.5% developed drug-related pneumonitis with no fatalities [66,68]. 

More recently, there has been an expansion of drug indications across tumour types based on shared receptor biology between different tumour types. For example, T-DXd is now approved for HER2-positive metastatic gastric and lung cancers [69,70]. In patients with metastatic gastric or gastro-oesophageal junction cancers with disease progression after two or more lines of previous therapy, T-DXd was associated with a 41% improvement in median overall survival compared to chemotherapy (HR 0.59, 95% CI 0.39 to 0.88) [69]. Multiple trials are ongoing using T-DXd in a wide variety of other HER2-positive malignancies. Another similar ADC is disitamab vedotin, which consists of a HER2-directed monoclonal antibody, cleavable linker, and MMAE payload [78]. There have been promising results with disitamab vedotin in a single-arm phase II trial of patients with HER2-positive advanced urothelial carcinoma, with an objective response rate of 51.2% [78].

Sacituzumab govitecan consists of a Trop-2-directed antibody linked via a cleavable linker to SN-38, a topoisomerase 1 inhibitor payload [72,74]. Trop-2 is an antigen that has been found to be over-expressed in triple-negative breast cancer and many other solid malignancies, and is associated with cancer progression and poor prognosis [94]. Sacituzumab govitecan has been shown to improve overall survival for patients with late-line metastatic triple-negative breast cancer compared to the treatment of a physician’s choice (HR 0.48, 95% CI 0.38 to 0.59; *p* < 0.001) [72]. More recently, it has also been demonstrated to improve PFS in patients with metastatic hormone receptor-positive breast cancer compared to treatment of physicians choice (HR 0.66, 95% CI 0.53–0.83; *p* = 0.0003) [71]. Furthermore, sacituzumab govitecan has shown clinical benefit in a single-arm phase II trial of patients with metastatic urothelial cancer with progressive disease after platinum and immunotherapy [73,95,96]. There are currently at least 19 trials underway studying the use of sacituzumab govitecan in a range of malignancies, including glioblastoma and refractory metastatic epithelial cancers, and breast, non-small cell lung, urothelial, prostate, head and neck, endometrial, and ovarian cancers [97]. 

Enfortumab vedotin combines a nectin-4-directed monoclonal antibody linked via a cleavable linker to MMAE [75,76,77]. Despite having a different antibody target, linker, and payload to sacitizumab govitecan, it has shown similar benefits in patients with metastatic urothelial cancer who have disease progression after platinum and immunotherapy. In the phase III trial in patients with progressive disease after platinum and immunotherapy, enfortumab vedotin was shown to improve overall survival compared with chemotherapy (HR 0.70; 95% CI 0.56 to 0.89; *p* = 0.001) [77].

Two ADCs have been recently approved for refractory advanced gynaecological cancers based on data from phase II trials. Mirvetuximab soravtansine is composed of a folate receptor alpha (FRα) antibody, a cleavable linker, and DM4 payload, another microtubule inhibitor [80]. FRα is a cell surface glycoprotein that mediates various cellular processes such as cell division, proliferation, and tissue growth [98]. It is over-expressed in over 90% of ovarian cancers, as well as in uterine, lung, and breast cancers [98]. A phase II single-arm trial evaluated mirvetuximab soravtansine in patients with platinum-resistant ovarian cancer with high FRα expression who had disease progression after 1–3 lines of chemotherapy plus bevacizumab. ORR was 32.4%, median PFS of 5.5 months, and median overall survival of 13.8 months [80]. In comparison, the standard of care for platinum-resistant recurrent ovarian cancer of chemotherapy plus bevacizumab is associated with response rates of 27.3% and a median PFS of 6.7 months [99]. Similarly, outcomes are poor for those with metastatic cervical cancer who have progressive disease after first-line therapy. Tisotumab vedotin is an ADC directed against tissue factor, with a cleavable linker and DM4 payload [79]. Tissue factor is physiologically expressed on adventitial cells and released after endothelial injury; however, it is also pathologically over-expressed on the surface of tumour cells and endothelial cells in various cancers, including pancreatic cancer, cervical cancer, sarcoma, lung cancer, triple-negative breast cancer, and acute lymphocytic leukaemia [100]. A phase II single-arm trial of tisotumab vedotin in patients with disease progression on or after doublet chemotherapy with bevacizumab demonstrated an ORR of 24%, median PFS of 4.2 months, and median overall survival of 12.1 months [79]. The confirmatory phase III trials of mirvetuximab soravtansine (clinicaltrials.gov identifier NCT04209855) and tisotumab vedotin (clinicaltrials.gov identifier NCT04697628) are ongoing [101]. 

### 4.2. ADCs in Haematological Malignancies

There are multiple ADCs approved for haematological malignancies, as illustrated in Table 2. The first ADC to be approved by the FDA was gemtuzumab ozogamicin for the treatment of adults with relapsed CD33+ acute myeloid leukaemia [6]. Gemtuzumab ozogamicin is a CD33-targeted ADC with a cleavable linker and calicheamicin payload [85]. Approval was subsequently withdrawn after the phase III SWOG S0106 trial demonstrated a higher mortality rate of 5.5% and high rates of hepatic toxicity, with gemtuzumab ozogamicin (6 mg/m^2^) plus standard chemotherapy compared to 1.4% with standard chemotherapy alone [102]. Later, randomised phase III clinical trials using a lower dose of gemtuzumab ozogamicin of 3 mg/m^2^ showed clinical benefit and improved safety, which led to its re-approval by the FDA in 2017 [81,82,83,84,85,86]. Inotuzumab ozogamicin, which targets CD22 with a cleavable linker and calicheamicin payload, has been associated with improved overall survival (HR 0.74, 97.5% CI 0.57–0.99, *p* = 0.01) in patients with relapsed or refractory B-cell precursor acute lymphoblastic leukaemia compared to treatment of physician’s choice [91]. Moxetumomab pasudotox which also targets CD22 with the same cleavable linker as inotuzumab ozogamicin, but with a different payload (pasudotox), has shown benefit in a small single-arm phase II trial in patients with relapsed or refractory hairy cell leukaemia, with a median PFS of 41.5 months [92]. 

Brentuximab vedotin, which targets CD30 with a cleavable linker and MMAE payload, has been approved for several haematological malignancies [62,87,88,89]. In the phase III ECHELON-1 trial of untreated stage III-IV classical Hodgkin lymphoma, brentuximab vedotin with doxorubicin, vinblastine, and dacarbazine was compared to doxorubicin, bleomycin, vinblastine, and dacarbazine. A 5-year PFS was improved with the addition of brentuximab vedotin (HR 0.68, 95% CI 0.53–0.87, *p* = 0.0017) [88]. It has also been shown to improve 5-year overall survival in CD30 expressing peripheral T cell lymphoma when added to cyclophosphamide, doxorubicin, and prednisone compared to cyclophosphamide, doxorubicin, vincristine, and prednisone (HR 0.72, 95% CI 0.53–0.99) [87]. Similarly, in a small phase III trial of relapsed primary cutaneous anaplastic large cell lymphoma or CD30 expressing mycosis fungoides, brentuximab has shown improved response rates and PFS [89]. 

Polatuzumab vedotin targets CD79b, possesses a cleavable linker and MMAE payload [90]. In a small phase Ib/II trial of patients with relapsed or refractory diffuse large B cell lymphoma, when combined with bendamustine and rituximab, it was shown to improve OS, compared to bendamustine and rituximab alone (HR 0.42, 95% CI 0.24 to 0.75, *p* = 0.002) [90]. Similarly, loncastuximab tesirine, which targets CD19, has a cleavable linker and pyrrolobenzodiazepine dimer payload, has been shown in a small single-arm phase II trial of patients with relapsed or refractory large B cell lymphoma to be associated with a high response rate of 48.3% and median overall survival of 9.9 months [93]. In addition, belantamab mafodotin which targets B cell maturation antigen, and possesses a non-cleavable linker and MMAF payload, has been shown to be active in a small phase II trial in a heavily pre-treated population of patients with multiple myeloma, with a 31–34% response rate depending on the dose used [25]. 

## 5. Challenges in the Clinical Development of ADCs and Limitations of Current ADCs

ADCs continue to gain popularity owing to heightened efficacy compared to conventional chemotherapy. Despite this, limitations exist, with over 50 potential ADCs having ceased development due to barriers such as limited efficacy or toxicity [103]. Early-phase research into ADCs poses distinct challenges and requires different approaches to trials of conventional cytotoxic therapy. For instance, phase I trials have traditionally been designed to find the maximum tolerated dose (MTD) of drugs on the basis that toxicity and response are positively correlated. However, in trials of targeted therapies and immunotherapies, it has been shown that there is not a predictable linear correlation between dose and efficacy [104]. This relationship has not been well studied in ADC trials. Still, it is likely to be distinct from traditional cytotoxic chemotherapy since various factors other than dose can influence ADC efficacy and therapeutic windows. These factors include the homogeneity or heterogeneity and level of expression of the target antigen on the tumour tissue, the degree of expression of target antigen on normal tissues, premature extracellular deconjugation of ADCs due to linker instability, and the permeability of the payload across cell membranes, and, hence, the degree to which the bystander effect occurs [105]. A threshold effect can exist for ADCs, whereby exceeding a particular dose of an ADC does not increase exposure or efficacy. There are various strategies that have been suggested to improve the optimal dose delivery of ADCs. These include body weight dose capping, treatment duration capping, altering the dose frequency, response-guided dosing, and randomised dose-finding studies [105]. Further improvements in the design and dose-finding of early-phase trials using ADCs are greatly needed.

Toxicities resulting from off-target effects where the payload is released to other tissues represent another obstacle to the development and adoption of ADCs. These are typically hepatic, neurologic, haematologic, respiratory or ophthalmic in nature [106]. Key examples of toxicities include the expression of HER2 and Nectin-2 on cardiomyocytes and skin, causing cardiotoxicity and skin toxicity, respectively [107]. Due to the unique makeup of different ADCs, adverse effect profiles vary and may be unique to the ADC, highlighting the importance of being able to provide reference guides for the individual drugs [108]. Early in ADC development, premature release of ADC payloads causing higher toxicity was associated with linker instability. To reduce this, the half-life of the ADC needed to be 10 times that of the payload itself [109]. Another key parameter to minimise premature drug release and toxicity is the polarity of the linker. This balance enhances payload coupling and reduces immunogenicity, while maintaining an appropriate payload delivery [110,111]. If the cytotoxic agent is too hydrophobic, this can change the antibody properties, leading to aggregation or conjugation. These balances can influence drug efficacy and tolerability [55]. Neutropaenia is a common and important toxicity of ADCs, particularly of MMAE-based ADCs with valine-citrulline linkers. With ADCs that contain a valine-citrulline linker and MMAE payload, neutropaenia occurs because neutrophils in the bone marrow produce serine proteases, which subsequently cleave the valine-citrulline linker and lead to the premature extracellular release of MMAE, and neutrophil death [112]. Another potential limitation of ADCs is in cancers that possess a dense tumour stroma. High-molecular weight drugs, including ADCs, have limited ability to penetrate dense tumour stroma to reach the required target [51]. 

### Mechanisms of Resistance to ADCs

Understanding resistance mechanisms to ADCs is an emerging area that needs further research. The areas of weakness that cancer can exploit include the internalisation process, payload mechanism, and the interaction between the antigen and antibody. Despite the potency of the payload, resistance to this can occur [113]. Contributing factors can be the ABC transporters (drug efflux pumps), historically known to impact and decrease the effectiveness of chemotherapeutic agents [113]. Preclinical in vitro models of breast cancer cells exhibiting ABCC1 (multidrug resistance protein 1) expression exhibited 256-fold increased resistance to T-DM1 after three months of cyclical treatment [114]. Cancer cells exposed to chronic unconjugated tubulin inhibitors administration can also induce the drug transporter MDR1 which is hypothesised to play a role in DM1 resistance [113]. This transporter, along with MRP1, can be upregulated from chronic exposure leading to acquired resistance, efflux upregulation, and drug deposition [113]. Acquired or intrinsic resistance is a challenge of ADCs, including antibody resistance, inability to traffic the required drug, lysosomal dysfunction, and payload inefficiency [113]. Currently, no resistance models correlating loss of ADC activity and conjugate internalisation exist. Proteomic profiling has shown utility in identifying protein alteration involving different aspects of the internalisation process, including lysosome biogenesis, vehicle transport, the cytoskeleton, and trafficking of the antibody [114]. Loss of lysosomal transporters could also decrease ADC efficacy [114,115].

ADC payload resistance and challenges are seen in a variety of cancer subtypes. An example includes a patient with long exposure to sacituzumab govitecan who underwent biopsies after death [116]. Tumour subclones with the mutation *TOP1*, known to encode topoisomerase-1 and the mutation *TACSTD2*, the encoder for *TROP2*, were found [116]. Parallel resistance mechanisms could occur affecting payload and antibody concurrently [116]. HER-2 receptor kinase or kinase signalling pathway alteration is a resistance mechanism after persistent ADC exposure. The T-DM1 resistant preclinical model KPL-4-T-DM1-R demonstrated decreased levels of HER2 and HER3 while other kinases such as EGFR increased [117]. Antigen expression and heterogeneity have been shown to be a mechanism of resistance in preclinical studies as described in the JIMT1 lines (resistant cell lines), where xenograft tumours treated with T-DM1 exhibited lower HER2 expression, which is associated with higher relapse rates and lower survival rates [118]. Changed target expression, before or during treatment, was associated with potentially worse outcomes in haematological malignancies, including myeloid leukaemia with low CD33 expression [113,119].

## 6. Future Directions

There is a substantial amount of research being conducted into ADCs, with over one hundred ADCs in preclinical and early-stage clinical research [6]. While historically targeted therapies have targeted oncogenic driver mutations, given how efficacious ADCs have been, we are seeing a shift in designs of ADCs so that the antigenic target is not necessarily an oncogenic driver but rather simply a target that is preferentially over-expressed in malignant cells. Haematological malignancies have more identifiable targets due to lineage-specific antigens making them the perfect candidates to target, while antigenic targets are often more heterogeneously expressed and less specific in solid organ tumours [120,121].

### 6.1. Developing Novel Antigenic Targets and Antibodies

Some of these new antigenic targets that are being explored in solid tumours include prostate-specific membrane antigen (PSMA), a six-transmembrane epithelial antigen of prostate-1 (STEAP-1), tissue factor, delta-like protein 3 (DLL-3), mesothelin, ENPP3 and B7-H3 family of proteins [122]. DLL-3 is an inhibitory Notch pathway ligand that mediates oncogenesis in melanoma, bladder, endometrial, ovarian, pancreatic, and lung cancer via multiple mechanisms, including angiogenesis, tumour stromal remodelling, and effects on immune cells in the tumour stroma [123]. PSMA is a membrane glycoprotein that is highly and selectively expressed in prostate cancer, and a PSMA ADC using an MMAE payload and valine-citrulline dipeptide linker has shown safety and activity in phase I trials [124]. STEAP-1 is a cell membrane protein that acts as an ion channel or transporter protein and is highly expressed in prostate, breast, pancreas, bladder, gastrointestinal tract, testicular, ovarian, and cervical cancers, Ewing sarcoma, and melanoma [125]. DSTP3086S is an ADC that consists of a humanized IgG1 linked through a protease cleavable linker to MMAE and has demonstrated in a phase I trial to have acceptable safety and evidence of activity in metastatic castrate-resistant prostate cancer [126].

A phase I trial of an ADC targeted against ENPP3, a protein expressed by most clear cell renal cell carcinomas, has reported tolerable toxicity and efficacy [127]. B7-H3 is an immune checkpoint protein that is overexpressed in many paediatric cancers, as well as non-small cell lung cancer and prostate cancer [128]. B7-H3 ADCs are currently being studied in medulloblastoma, peritoneal cancer, neuroblastoma, glioma, prostate cancer, head and neck cancer, non-small cell lung cancer, urothelial cancer, rhabdomyosarcoma, osteosarcoma, Ewing sarcoma, and Wilms’ tumour [128]. For instance, AbBV-155 (mirzotamab clezutoclax), an anti-B7-H3 ADC, has been evaluated in non-small cell lung cancer and breast cancer. No significant dose-limiting toxicities were reported in the single agent phase one cohort, with a partial response occurring in 21% of patients [129]. Mesothelin is a cell membrane glycoprotein that is expressed in mesothelioma, lung adenocarcinoma, pancreatic adenocarcinoma, colorectal adenocarcinoma, serous ovarian cancer, gastric adenocarcinoma, and breast cancer [130]. Various other ADCs targeting mesothelin are currently in development [130].

Various other targets are being explored for haematological malignancies, including CD37 for patients with relapsed and refractory diffuse large B cell lymphoma with naratuximab emtansine, CD138 with indatuximab ravtansine for multiple myeloma, CD19 with coltuximab mertansine for diffuse large B cell lymphoma and acute lymphocytic leukaemia, CD56 with lorvotuzumab mertansine for CD56 expressing haematological malignancies, and CD22 with pinatuzumab vedotin for diffuse large B cell lymphoma and follicular non-Hodgkin lymphoma [131].

ADCs employing bispecific antibodies are being explored as a potential means of improving efficacy in tumours with heterogeneous antigen expression. Bispecific ADCs can target two different antigens or different sites on the same antigen. Targeting two different sites on the same antigen is thought to enable more efficient internalisation of the compound and improve receptor aggregation [6]. For example, ADCs with bispecific antibodies targeting HER-2 and the prolactin receptor have been shown to improve ADC internalisation and have higher anti-tumour activity in vitro compared to a conventional HER2-directed ADC [132].

There is also increasing research into targeting cells in the tumour microenvironment. Cancer-associated fibroblasts are thought to promote therapeutic resistance and promote cancer cell survival. Two novel ADCs, fibroblast activation protein α monoclonal antibody conjugated to DM1 and fibroblast activation protein α conjugated to pseudomonas exotoxin 38, have been shown to be highly effective in xenograft models of lung, head and neck, pancreatic and breast cancers [133,134].

### 6.2. Development of Improved Cytotoxic and Other Payloads

Novel payloads and payload structures are also being explored. Dual payloads are being explored to improve responsiveness in solid tumours with heterogeneous target expression. Newer payloads in development include pyrrolobenzodiazepine monomers or dimers, indolino–benzodiazepines, and cyclopropabenzindolone monomers and dimers, with IC_50_ values in the picomolar range. Some have been hindered by high rates of toxicity [16].

For instance, a HER2-targeted ADC containing dual payloads of MMAE and MMAF was designed and tested in a xenograft model of HER2+ breast cancer. The dual payload containing ADC was highly effective at killing tumour cells in vivo, more so than when both single payload-based ADCs were used together [135]. Other payloads in development include BCL-XL inhibitors that can induce apoptosis selectively in tumours that are BCL-XL dependent [136,137]. Overexpression of BCL-XL is often seen in cancers such as melanoma and glioblastoma [138].

Tyrosine kinase inhibitors have also been studied as payloads, as kinase families are known to be heavily involved in cell cycle progression, proliferation, angiogenesis, and movement of cells around the body. At present, tyrosine kinase inhibitor-based ADCs have not been as efficacious as hoped [16]. Tyrosine kinase inhibitor plus ADC combinations are being investigated to offset tumour heterogeneity and resistance. For instance, T-DM1 and tucatinib, a HER2-selective tyrosine kinase inhibitor, have been used in combination. This demonstrated an objective response rate of 47% in patients previously treated with trastuzumab and a prior taxane, along with a brain-specific response of 36% [139]. Recruitment into this specific combination is ongoing [139]. Photoimmunotherapy is another emerging treatment whereby monoclonal antibodies are conjugated with a light-activated dye, which when activated, disrupts tumour cells, leading to cell death. For example, cetuximab sarotalocan combines an EGFR monoclonal antibody with the light activatable dye, IR700, and in a phase I trial of three patients with recurrent head and neck squamous cell carcinoma, two of three patients experienced a response with a manageable safety profile [140].

### 6.3. Immunotherapy and ADCs

There is increasing research into developing ADCs that have a heightened ability to stimulate the immune system, which is particularly relevant as ADCs work partly through Antibody-dependent cellular cytotoxicity. Research is ongoing into two approaches: first, using immunotherapy in combination with ADCs, and second, incorporating immunotherapy into ADCs. ADCs interact with the local tumour immune microenvironment via activation of dendritic cells, activation of T cells, and upregulation of damage-associated molecular patterns (DAMPs) and have been shown to enhance the anti-tumour effect of immunotherapy in preclinical models [141,142]. Combining HER2-directed ADCs and immunotherapy was trialled in the phase II KATE2 study, where T-DM1 and atezolizumab were compared to T-DM1 and placebo in pre-treated patients with HER-2-positive breast cancer. The combined immunotherapy and HER2 treatment failed to improve PFS, but a trend was noted for benefit in those with PDL-1 expression [143]. Despite this combination not having the desired outcome, combining ADC and immunotherapy in early-phase studies is promising in a plethora of cancers, including small cell lung, ovarian, triple-negative breast cancer, and urothelial cancer [8].

In the second approach, ADCs are designed to stimulate the immune system. The two main categories of these immune-stimulating ADCs in development at present are ADCs containing STING agonists and TLR agonists [16]. Conventional STING and TLR agonists have been unsuccessful to date owing to high rates of toxicity, particularly characterised by cytokine release syndrome. The first immunostimulatory ADC to reach clinical trials, NJH395, combines a small molecule TLR7/8 agonist with an anti-HER2 monoclonal antibody. However, results were disappointing in the phase I clinical trial of 18 patients with non-breast HER2-positive malignancies, characterised by high rates of cytokine release syndrome and limited efficacy [144,145].

In summary, ADCs represent a new class of therapies that combines the strengths that therapeutic antibodies and potent chemotherapy deliver. While there has been significant success with ADCs recently, the field remains in its infancy. Multiple areas have not yet been thoroughly studied, such as resistance mechanisms, the optimal dosing of ADCs, and the interplay between the immune system and ADCs. As more ADCs come into clinical use, recurrent themes on its mechanisms of action and toxicities are likely to emerge, although each ADC is likely to be unique in its own right due to its combination of antibody, payload, and linker, with significant opportunities existing to improve upon each component.

## Figures and Tables

**Table 1:** Positive phase II/III trials of antibody–drug conjugates in solid tumours leading to FDA approval.

Drug	FDA Approval	Pivotal Trial(s)	Population	Number of Patients	Antibody Target, Linker and Payload	Results of Intervention vs. Comparator
**Trastuzumab emtansine** **(T-DM1)**	2013	EMILIA [64] (phase III)	Advanced HER2+ breast cancer with PD after trastuzumab + taxane.	T-DM1: 495 Capecitabine + lapatinib: 496	**Ab target:** HER2 **Linker:** SMCC (non-cleavable) **Payload:** DM1	ORR 43.6% vs. 30.8%, mPFS 9.6 vs. 6.4 mths, mOS 30.9 vs. 25.1 mths.
	2019	KATHERINE [65] (phase III)	Early-stage HER2+ breast cancer with residual disease after NACT.	T-DM1: 743 Trastuzumab: 743		3 yr iDFS 88.3% vs. 77.0%.
**Trastuzumab deruxtecan** **(T-DXd)**	2022	DESTINY-Breast03 [66] (phase III)	Advanced HER2+ breast cancer with PD after trastuzumab + taxane.	T-DXd: 261 T-DM1: 263	**Ab target:** HER2 **Linker:** GGFG tetrapeptide (cleavable) **Payload:** Deruxtecan	ORR 79.7% vs. 34.2%, mPFS not reached vs. 6.8 mths with T-DM1, mOS both not reached.
	2022	DESTINY-Breast02 [67] (phase III)	Advanced HER2+ breast cancer with PD after T-DM1.	T-DXd: 406 TPC: 202		ORR 70% vs. 29%, mPFS 17.8 vs. 6.9 mths, mOS 39.2 vs. 26.5 mths.
	2022	DESTINY-Breast04 [68] (phase III)	Advanced HER2 low breast cancer with PD after 1–2 lines of chemotherapy.	T-DXd: 373 TPC: 184		ORR 52.3% vs. 16.3%, mPFS 9.9 vs. 5.1 mths, mOS 23.4 vs. 16.8 mths.
	2021	DESTINY-Gastric01 [69] (phase II)	Advanced HER2+ gastric/GOJ cancers after ≥2 lines of therapy.	T-DXd: 125 TPC: 62		ORR 51% vs. 14%, mPFS 5.6 vs. 3.5 mths, mOS 12.5 vs. 8.4 mths.
	2022	DESTINY-Lung01 [70] (phase II)	Advanced HER2+ NSCLC refractory to standard therapy.	T-DXd: 91 (single arm)		ORR 55%, mPFS 8.2 mths, mOS 17.8 mths.
**Sacituzumab govitecan** **(SG)**	2023	TROPiCS-02 [71] (phase III)	Advanced HR+ breast cancer, HER2- or low with PD after ET and ≥2 systemic therapies.	SG: 272 TPC: 271	**Ab target:** Trop-2 **Linker:** CL2A (cleavable) **Payload:** SN-38	ORR 21% vs. 14%, mPFS 5.5 vs. 4.0 mths, mOS 13.9 vs. 12.3 mths.
	2020	ASCENT [72] (phase III)	Advanced TNBC with PD after ≥2 lines of chemotherapy.	SG: 235 TPC: 233		ORR 35% vs. 5%, mPFS 5.6 vs. 1.7 mths, mOS 12.1 vs. 6.7 mths.
	2021	TROPHY [73] (phase II)	Advanced urothelial cancer with PD after platinum and immunotherapy.	SG: 113 (single arm)		ORR 27%, mPFS 5.4 mths, mOS 10.9 mths.
	2020	IMMU-132-01 [74] (phase I/II)	Advanced TNBC after ≥2 lines of chemotherapy.	SG: 108 (single arm)		ORR 33.3%, mPFS 5.5 mths, mOS 13.0 mths.
**Enfortumab vedotin** **(EV)**	2019	EV-201 [75,76] (phase II)	Advanced urothelial carcinoma. **Cohort 1:** PD after platinum + immunotherapy. **Cohort 2:** PD after immunotherapy, no prior platinum.	**Cohort 1:** 125 **Cohort 2:** 89 (single arm)	**Ab target:** Nectin-4 **Linker:** mc-VC-PABC (cleavable) **Payload:** MMAE	**Cohort 1:** ORR 44%, mPFS 5.8 mths, mOS 11.7 mths **Cohort 2:** ORR 52%, mPFS 5.8 mths, mOS 14.7 mths.
	2019	EV-301 [77] (phase III)	Advanced urothelial carcinoma with PD after platinum and immunotherapy.	EV: 301 TPC: 307		ORR 40.6% vs. 17.9%, mPFS 5.6 vs. 3.7 mths, mOS 12.9 vs. 9.0 mths.
Disitamab vedotin * **(DV)**	2021	[78] (phase II)	Advanced HER2+ urothelial carcinoma with PD after ≥1 prior therapy.	DV: 43 (single arm)	**Ab target:** HER2 **Linker:** mc-VC-PABC (cleavable) **Payload:** MMAE	ORR 51.2%, mPFS 6.9 mths, mOS 13.9 mths.
**Tisotumab vedotin** **(TV)**	2021	InnovaTV 204 [79] (phase II)	Recurrent/advanced cervical cancer with PD after ≤2 lines of chemotherapy.	TV: 102 (single arm)	**Ab target:** tissue factor **Linker:** mc-VC-PABC (cleavable) **Payload:** MMAE	ORR 24%, mPFS 4.2 mths, mOS 12.1 mths.
**Mirvetuximab soravtansine (MIRV)**	2022	SORAYA [80] (phase II)	FRα high platinum-resistant ovarian cancer with ≤3 prior systemic therapies, including bevacizumab.	MIRV: 106 (single arm)	**Ab target:** FRα **Linker:** disulfide hydrophilic sulfo-SPDB (cleavable) **Payload:** DM4	ORR 32.4%, mPFS 4.3 mths, mOS 13.8 mths.

Abbreviations: Ab, antibody; ABVD, doxorubicin, bleomycin, vinblastine, and dacarbazine; ALL, acute lymphoblastic leukaemia; AML, acute myeloid leukaemia; AVD, doxorubicin, vinblastine, and dacarbazine; BCMA, B-cell maturation antigen; BG, bendamustine and obinutuzumab; BR, bendamustine and rituximab; BSC, best supportive care; CHOP, cyclophosphamide, doxorubicin, vincristine, and prednisone; CHP, cyclophosphamide, doxorubicin, and prednisone; CL2A, cross-linked 2A; CR, complete response; DLBCL, diffuse large B cell lymphoma; EFS, event-free survival; FRα, folate receptor α; GGFG, Gly-Gly-Phe-Gly; HR, hormone receptor; mc-VC-PABC, maleimidocaproyl-valyl-citrullinyl-p-aminobenzyloxycarbonyl; iDFS, invasive disease free survival; MMAE/F, monomethyl auristatin-E/F; mths, months; mPFS, median progression free survival; mOS, median overall survival; NACT, neoadjuvant chemotherapy; NMPA, National Medical Products Administration of China; ORR, objective response rate; PBD, pyrrolobenzodiazepine; PD, progressive disease; RFS, relapse-free survival; SG, sacituzumab govitecan; SMCC, succinimidyl-4-(N-maleimidomethyl) cyclohexane-1-carboxylate; SPDB, N-succinimydl 4-(2-pyridyldithio)−2-sulfobutanoate); TPC, treatment of physician’s choice. * Approved by NMPA.

**Table 2:** Positive phase II/III trials of antibody–drug conjugates in haematological malignancies leading to FDA approval.

Drug	FDA Approval	Pivotal Trial(s)	Population	Number of Patients	Antibody Target, Linker and Payload	Results of Intervention vs. Comparator
**Gemtuzumab ozogamicin (GO)**	2017	ALFA-0701 [81,82,83] (phase III)	Newly diagnosed, CD33+ AML, age 50–70.	GO + standard therapy: 140 SOC: 140	**Ab target:** CD33 **Linker:** hydrazone (cleavable) **Payload:** calicheamicin	2 yr EFS 40.8% vs. 17.1%, RFS 50.3% vs. 22.7%.
	2017	AAML0531 [84] (phase III)	Newly diagnosed AML age 0–29 years.	GO + standard therapy: 511 SOC: 511		3 yr EFS 53.1% vs. 46.9%, 3 yr OS 69.4% vs. 65.4%.
	2017	AML-19 [85] (phase III)	Newly diagnosed AML, >75 yrs or 61–75 yrs and unfit for intensive chemotherapy.	GO: 118 BSC: 119		mOS 4.9 vs. 3.6 mths.
	2017	MyloFrance-1 [86] (phase II)	CD33+ AML in first relapse.	GO: 57 (single arm)		ORR 33.3%, mOS 8.4 mths, mRFS 11.0 mths.
**Brentuximab vedotin** **(BV)**	2018	ECHELON-2 [87] (phase III)	Untreated CD30+ peripheral T cell lymphomas.	BV + CHP: 226 CHOP: 226	**Ab target:** CD30 **Linker:** mc-VC-PABC (cleavable) **Payload:** MMAE	5 yr PFS 51.4% vs. 43.0%, 5 yr OS 70.1% vs. 61.0%.
	2018	ECHELON-1 [88] (phase III)	Untreated stage III-IV classical Hodgkin lymphoma.	BV + AVD: 664 ABVD: 670		5 yr PFS 82.2% vs. 75.3%, OS immature.
	2017	ALCANZA [89] (phase III)	Relapsed primary cutaneous anaplastic large cell lymphoma or CD30+ mycosis fungoides.	BV: 64 TPC: 64		ORR 54.7% vs. 12.5%, mPFS 16.7 vs. 3.5 mths, 3 year OS 64.4% vs. 61.9%.
**Polatuzumab vedotin (PV)**	2019	Study GO29365 [90] (phase Ib/II)	Relapsed or refractory DLBCL with ≥2 prior therapies.	1. PV + BG: 20 2. PV + BR: 40 3. BR: 40	**Ab target:** CD79b **Linker:** mc-VC-PABC (cleavable) **Payload:** MMAE	**Phase I:** PV + BG mOS 10.8 mths. **Phase II:** PV + BR vs. BR mPFS 12.4 vs. 4.7 mths.
**Belantamab mafodotin (BM)**	2020	DREAMM-2 [25] (phase II)	Relapsed or refractory multiple myeloma with ≥4 prior therapies.	**Cohort 1** (BM 2.5 mg/kg): 97 **Cohort 2** (BM 3.4 mg/kg): 99	**Ab target:** BCMA **Linker:** mc (non-cleavable) **Payload:** MMAF	**Cohort 1:** ORR 31%, mPFS 2.9 mths. **Cohort 2:** ORR 34%, mPFS 4.9 mths.
**Inotuzumab ozogamicin (InO)**	2017	INO-VATE [91] (phase III)	Relapsed or refractory B-cell precursor ALL.	InO: 164 TPC: 162	**Ab target:** CD22 **Linker:** hydrazone (cleavable) **Payload:** calicheamicin	mOS: 7.7 vs. 6.2 mths, 2 yr OS: 22.8% vs. 10.0%.
**Moxetumomab** **pasudotox (MP)**	2018	Study 1503 [92] (phase II)	Relapsed or refractory hairy cell leukaemia.	MP: 80 (single arm)	**Ab target:** CD22 **Linker:** hydrazone (cleavable) **Payload:** pasudotox	Durable CR rate of 36%, median CR duration 62.8 mths, mPFS 41.5 mths.
**Loncastuximab tesirine** **(LT)**	2021	LOTIS-2 [93] (phase II)	Relapsed or refractory DLBCL after ≥2 therapies.	LT: 145 (single arm)	**Ab target:** CD19 **Linker:** valine–alanine (cleavable) **Payload:** PBD dimer	ORR 48.3%, mPFS 4.9 mths, mOS 9.9 mths.

Abbreviations: Ab, antibody; ABVD, doxorubicin, bleomycin, vinblastine, and dacarbazine; ALL, acute lymphoblastic leukaemia; AML, acute myeloid leukaemia; AVD, doxorubicin, vinblastine, and dacarbazine; BCMA, B-cell maturation antigen; BG, bendamustine and obinutuzumab; BR, bendamustine and rituximab; BSC, best supportive care; CHOP, cyclophosphamide, doxorubicin, vincristine, and prednisone; CHP, cyclophosphamide, doxorubicin, and prednisone; CL2A, cross-linked 2A; CR, complete response; DLBCL, diffuse large B cell lymphoma; EFS, event-free survival; FRα, folate receptor α; GGFG, Gly-Gly-Phe-Gly; HR, hormone receptor; mc-VC-PABC, maleimidocaproyl-valyl-citrullinyl-p-aminobenzyloxycarbonyl; MMAE/F, monomethyl auristatin-E/F; mth, months; mPFS, median progression free survival; mOS, median overall survival; mths, months; NACT, neoadjuvant chemotherapy; NMPA, National Medical Products Administration of China; ORR, objective response rate; PBD, pyrrolobenzodiazepine; PD, progressive disease; RFS, relapse-free survival; SOC, standard of care; SG, sacituzumab govitecan; SMCC, succinimidyl-4-(N-maleimidomethyl) cyclohexane-1-carboxylate; SPDB, N-succinimydl 4-(2-pyridyldithio)−2-sulfobutanoate); TPC, treatment of physician’s choice; yr, years.
